# Tuning phonon transport spectrum for better thermoelectric materials

**DOI:** 10.1080/14686996.2018.1548884

**Published:** 2018-12-21

**Authors:** Takuma Hori, Junichiro Shiomi

**Affiliations:** a Department of Mechanical Engineering, Tokyo University of Science, Noda, Japan; b Department of Mechanical Engineering, The University of Tokyo, Tokyo, Japan; c Center for Materials Research by Information Integration (CMI2), Research and Services Division of Materials Data and Integrated System (MaDIS), National Institute for Materials Science, Tsukuba, Japan; d CREST, Japan Science and Technology Agency, Kawaguchi, Japan

**Keywords:** Thermoelectric material, phonon transport, lattice thermal conductivity, alloy scattering, sintered nanostructure, particle resonance, 50 Energy Materials, 102 Porous / Nanoporous / Nanostructured materials, 210 Thermoelectronics / Thermal transport / insulators, 402 Multi-scale modeling

## Abstract

The figure of merit of thermoelectric materials can be increased by suppressing the lattice thermal conductivity without degrading electrical properties. Phonons are the carriers for lattice thermal conduction, and their transport can be impeded by nanostructuring, owing to the recent progress in nanotechnology. The key question for further improvement of thermoelectric materials is how to realize ultimate structure with minimum lattice thermal conductivity. From spectral viewpoint, this means to impede transport of phonons in the entire spectral domain with noticeable contribution to lattice thermal conductivity that ranges in general from subterahertz to tens of terahertz in frequency. To this end, it is essential to know how the phonon transport varies with the length scale, morphology, and composition of nanostructures, and how effects of different nanostructures can be mutually adopted in view of the spectral domain. Here we review recent advances in analyzing such spectral impedance of phonon transport on the basis of various effects including alloy scattering, boundary scattering, and particle resonance.

## GRAPHICAL ABSTRACT

**Figure:**
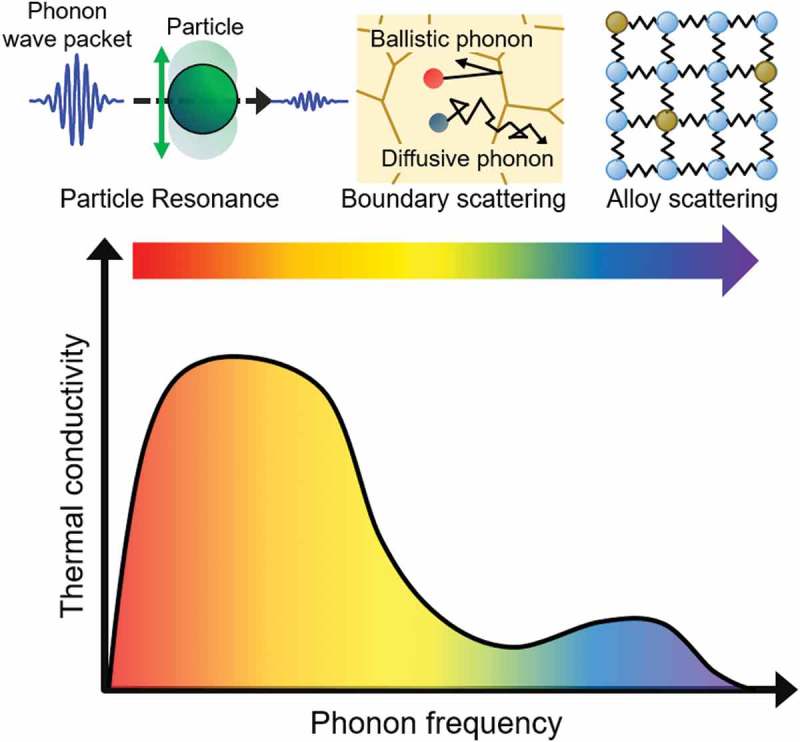

## 1. Introduction

Thermoelectrics has attracted a great attention as a way for energy harvesting or waste heat recovery. The conversion efficiency of thermoelectric materials is governed by the dimensionless parameter, *ZT* = *S*^2^*σT*/(*κ*_p_*+ κ*_e_) [1], where *S* is the Seebeck coefficient, *σ* is the electrical conductivity, *T* is the absolute temperature, and *κ*_p_ and *κ*_e_ are the lattice and electrical thermal conductivities. Therefore, the challenge for achieving high *ZT* is to enhance electrical properties and reduce lattice thermal conductivity simultaneously. The ideal thermoelectric materials is often referred to as *phonon-glass electron-crystal* [2], which is in general hard to achieve [3]. Great efforts have been made to enhance electrical properties by, for instance convergence of electronic bands [4], energy filtering [5,6], and resonance doping [7,8]. On the other hand, the reduction of the lattice thermal conductivity has been realized by controlling the properties of phonons. Phonons are the carriers for lattice thermal conduction [9], and the thermal conductivity in crystals can be characterized as the diffusion of phonons.

**Figure 1.**
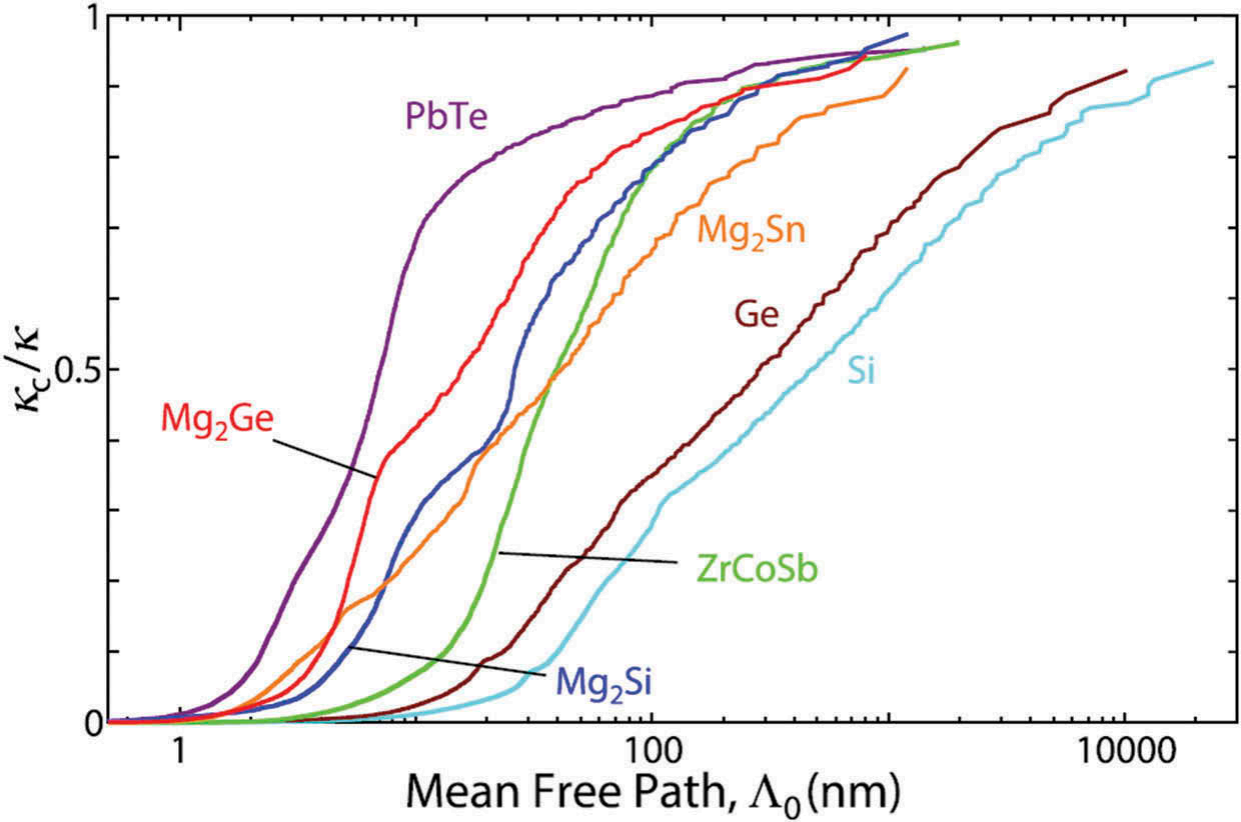
Dependence of cumulative lattice thermal conductivity *κ_c_* on MFP for various crystals calculated by ALD with first-principles force constants. The values are normalized by the bulk lattice thermal conductivity κ_p_ [13]. Reproduced with permission from [13]. Copyright 2015 AIP Publishing.

Phonons are the lattice vibration modes, and their states are described with wave vector **q** in reciprocal space and branch *s* the representing polarization. The frequency of phonon is uniquely determined as a function of the wave vector and branch, namely the phonon dispersion relations, which can be obtained from the knowledge of crystal structure and interatomic potential. A well-known theoretical model of the phonon dispersion relations is the Debye model, which can successfully reproduce the temperature dependence of specific heat at low temperatures observed in experiments by incorporating the spectral feature of phonon states [10]. The spectral feature of phonon must be also taken into account to accurately describe thermal conduction. From the Boltzmann transport equation, the lattice thermal conductivity can be expressed as follows:

(1) $$\kappa_{p}=\frac{1}{3}\underset{q,\;s}{\sum }C_{q,\;s}v_{q,\;s}^{2}\tau_{q,\;s}=\frac{1}{3}\underset{q,\;s}{\sum }C_{q,\;s}v_{q,\;s}\Lambda_{q,\;s},$$

where *C* is heat capacity per phonon mode, *v* is phonon group velocity, *τ* is relaxation time, and Λ = *vτ* is mean free path (MFP) [9]. These properties strongly depend on the mode of phonons; for instance, MFP is typically longer for lower frequency. The resulting mode-dependent contribution to lattice thermal conductivity is often quantified by the cumulative lattice thermal conductivity *κ_c_* defined in Equation (2) [11,12],

(2) $$\kappa_{c}\left(\Lambda_{0}\right)=\frac{1}{3}\overset{\Lambda_{q,\;s}\leq \Lambda_{0}}{\underset{q,\;s}{\sum }}c_{q,\;s}v_{q,\;s}\Lambda_{q,\;s},$$

where Λ_0_ is the threshold MFP. The cumulative lattice thermal conductivity is the summation of the lattice thermal conductivities by the phonons with MFPs shorter than Λ_0_. The examples of the cumulative lattice thermal conductivity of various materials, normalized by the bulk lattice thermal conductivity *κ*_p_ is shown in Figure 1 [13]. For instance, in silicon, *κ_c_*/*κ*_p_ is approximately 10% at Λ_0_ = 50 nm, and 90% at Λ_0_ = 10 μm at room temperature. As seen here, phonons with a wide range of MFPs noticeably contribute to lattice thermal conductivity, and this is why evaluation of the modal phonon properties is important, in particular for nano-structures whose length scale lies in the middle of the MFP range. Compared with specific heat and group velocity, evaluation of relaxation time is relatively challenging since the relaxation originates from anharmonic lattice dynamics. However, the recent progress in numerical simulation techniques, such as anharmonic lattice dynamics (ALD) method with first-principles interatomic force constants [14–18], has enabled us to access these anharmonic phonon transport properties. In addition to the numerical methods, experimental methods, such as inelastic neutron scattering [19,20], inelastic x-ray scattering [21,22], and thermoreflectance [23–26] methods have been developed to probe the modal phonon transport properties.

As stated above, for better performance of thermoelectrics, it is necessary to reduce the lattice thermal conductivity without degrading the electrical properties. Alloying is the traditional approach to realize this difficult balance. In a substitutive alloy or a solid solution, atoms or compounds are mixed in a single homogeneous phase keeping the original crystal structures. This approach mainly aims to facilitate phonon scattering by disordering vibration modes which reduces the relaxation time *τ*. The alloying has been extensively demonstrated and studied for many thermoelectric materials such as Si*_x_*Ge_1__−_*_x_* [27–29], Bi*_x_*Sb_2__−_*_x_*Te_3_ [30–33], Mg_2_Si*_x_*Sn_1__−_*_x_* [34–37], Bi*_x_*Sb_1__−_*_x_* [38], PbTe*_x_*Se_1__−_*_x_* [39,40], half-Heusler compounds [41–46], since half a century ago.

As for more recent approach, development in nanotechnology has realized fabrication of nanostructured materials for better thermoelectric performance [47]. Initially, nanostructuring was mainly carried out aiming to increase Seebeck coefficient by quantum confinement [48–50]. Recently, the main objective of nanostructuring has shifted to lowering the lattice thermal conductivity. The mechanism of the lattice-thermal-conductivity reduction by nanostructuring can be explained in the view of phonon transport; phonons experience boundary scattering at vast number of interfaces existing in nanostructures, which corresponds to shortening of MFP [51]. Among the early works on nanostructured thermoelectric materials, ones on super-lattice structures are notable because of the record *ZT* at that time [52,53]. Although it has been pointed out that the data have not been reproduced by other groups [54], it is true that the works have greatly stimulated the research field. Various nanostructures such as nanowires [55,56], sintered nanopolycrystals [57,58], nanoporous materials [59,60], and embedded nanoparticles [61–63] have been fabricated and demonstrated to exhibit high thermoelectric efficiency. Recently, the nanostructuring effect on the conversion efficiency has also been investigated in spin Seebeck thermoelectric materials [64].

**Figure 2.**
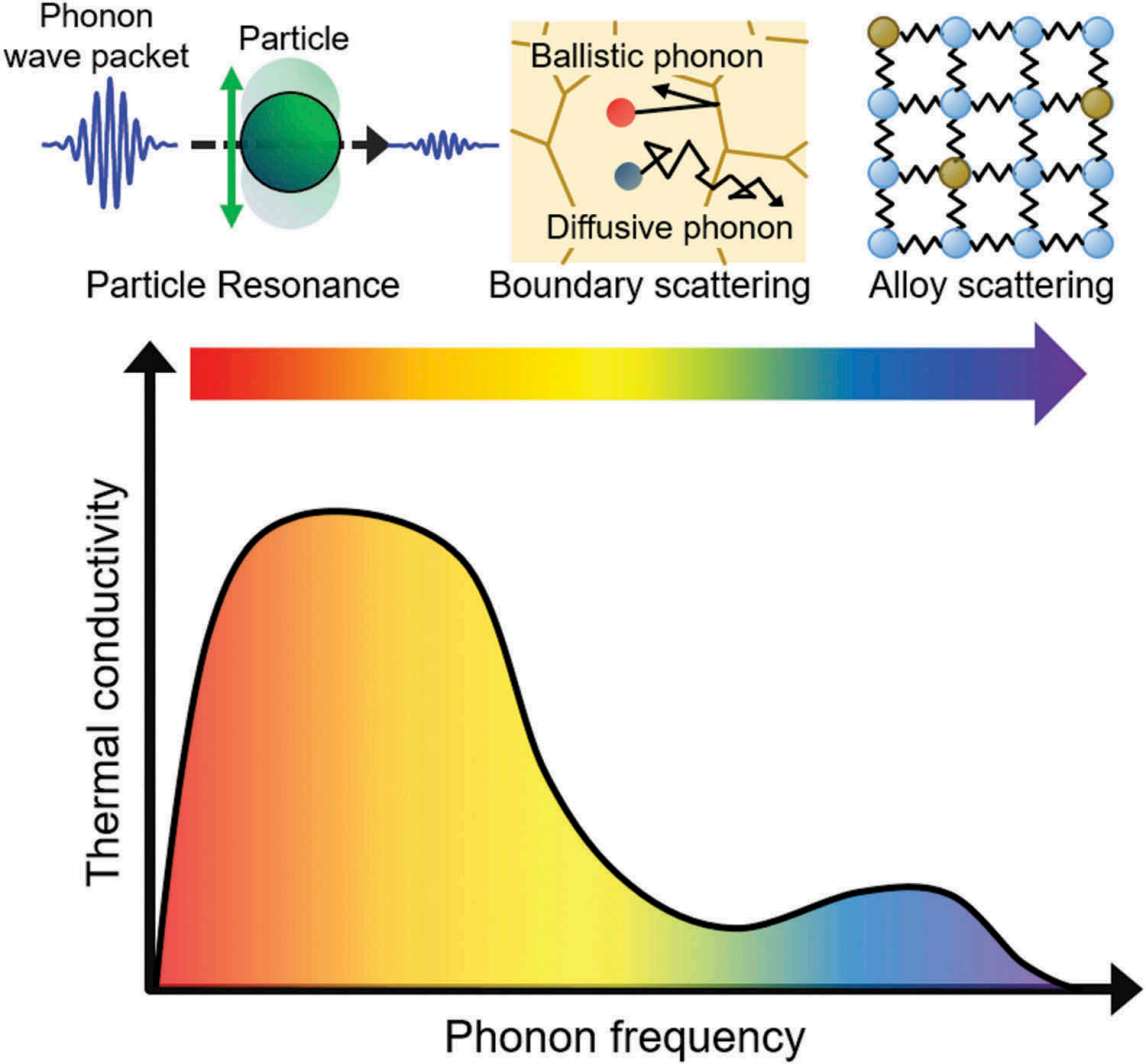
A schematic of frequency dependence of lattice thermal conductivity and the frequency range where different structures are effective in scattering phonons. Phonons with low, medium, and high frequency can be scattered by particle resonance, boundary scattering, and alloy scattering effects, respectively.

The combination of alloying and nanostructuring is beneficial to further reduce lattice thermal conductivity. For example, an experimental work utilizing the combination of the embedded nanoparticles, impurities, and sintered interfaces has achieved thermoelectric materials with high performance, which was attributed to the low lattice thermal conductivity [63]. It was suggested that these multiscale structures hierarchically scatter phonons of a wide range of modes. Figure 2 shows the schematic diagram in the frequency domain. As we will introduce in this review, the alloy and interfaces effectively scatter phonons with high frequency (short MFP) and low frequency (long MFP), respectively. Meanwhile, it is challenging to suppress the lattice thermal conductivity of phonons with extremely low frequencies in bulk materials. In addition, some higher frequency modes do remain to have noticeable contribution to thermal conductivity even after alloying and nanostructuring. Theoretical calculations show that such phonon modes can be additionally diminished via resonance effect by embedded nanoparticles, where in ideal case, the transport of specific phonon modes are selectively terminated.

The key point in designing material with low lattice thermal conductivity is to quantitatively understand transport of which phonon modes can be impeded by alloy scattering, boundary scattering, and particle resonance. This is especially important when it comes to the combination of different mechanisms to impede the transport of a broad range of phonon modes. While there have been many reviews on the related topics [65–83], this review particularly focuses on the lattice thermal conductivity reduction through alloy scattering, boundary scattering, and particle resonance in light of phonon spectral properties. In Section 2, recent analytical works on alloy effect on the lattice thermal conductivity and phonon transport are introduced. Section 3 reviews works on phonon transport in nanocrystals, including detailed description of how to analyze the phonon transport across the interfaces and in complex nanostructures. Section 4 focuses on the particle resonance effect that can impede the phonon transport of targeted modes. In what follows, we simply address ‘lattice thermal conductivity’ as ‘thermal conductivity’ since the focus here is on phonon transport.

## 2. Phonon-impurity scattering due to alloying

The mechanism of the lattice thermal conductivity reduction by alloying has been understood as phonon-impurity scattering. Once the phonon-impurity scattering rate *τ*_imp_^−1^ is obtained, the effective relaxation time *τ*_a_*_−_*_i_ can be calculated with intrinsic relaxation time *τ*_anh_ by utilizing the Matthiessen’s rule [84].

(3) $$\tau_{a-i}^{-1}\left(q,\;s\right)=\tau_{anh}^{-1}\left(q,\;s\right)+\tau_{imp}^{-1}\left(q,\;s\right).$$

Therefore, the challenge to evaluate spectral thermal conductivity of alloys lies in obtaining the mode-dependent scattering rates induced by different atomic species composing the alloys. A widely used model of the scattering rate, known as the Klemens model [85], estimates that the phonon impurity scattering rate is proportional to the fourth power of its frequency. Here the Klemens model assumes the linearized phonon dispersion relation (Debye approximation). Tamura model [86] based on perturbation theory was later derived, where the scattering rate is expressed by Equation (4) incorporating realistic phonon dispersion relation.

(4) $$\begin{array}{rl} & \tau_{tmr}^{-1}\left(q,\;s\right)=\frac{\pi }{2N}\omega^{2}\left(q,\;s\right)\underset{q^{\prime },\;s^{\prime }}{\sum }\delta \left[\omega \left(q,\;s\right)-\omega \left(q^{\prime },\;s^{\prime }\right)\right]\\ & \;\;\;\;\;\underset{\alpha }{\sum }g_{2}\left(\alpha \right){\left|e\left(\alpha ,q,\;s\right)|e\left(\alpha ,q^{\prime },\;s^{\prime }\right)\right|}^{2}, \end{array}$$

where *N* is the number of unit cells, **e** is the polarization vector, and

(5) $$g_{2}\left(\alpha \right)=\underset{i}{\sum }\;f_{i}\left(\alpha \right){\left[1-\frac{m_{i}\left(\alpha \right)}{\bar{m_{i}}\left(\alpha \right)}\right]}^{2},$$

Here, *f_i_*(*α*) and *m_i_*(*α*) are the mole fraction and relative atomic mass of the *i*th isotope of the *α* atom in unit cells, respectively. Equation (4) indicates that the scattering rates of high-frequency modes are moderate compared with Klemens model but they are still much higher than those of lower frequency modes. This model is widely used to predict the thermal conductivity of alloys, such as Si*_x_*Ge_1__−_*_x_* [87], PbTe*_x_*Se_1__−_*_x_* [88], Bi*_x_*Sb_1__−_*_x_* [89], Mg_2_Si*_x_*Sn_1__−_*_x_* [90], half-Heusler [91], Pb*_x_M*_1__−_*_x_*Te (*M* = Mg, Ca, Sr, Ba) [92] and even a phase transition material Pb*_x_*Ge_1__−_*_x_*Te [93] combining phonon transport properties of the bulk crystals obtained by ALD calculation with first-principles interatomic force constants. In these works, virtual crystal approximation [94] is employed to evaluate intrinsic phonon properties such as *τ*_anh_, where the mass and force field of the crystal are kept homogeneous but modulated to be the weighted average between the values of host and guest atoms or compounds. Note that this ‘ALD + Tamura’ model is rigorous for quantifying the effect of low-concentration isotopes on the thermal conductivity [95,96].

**Figure 3.**
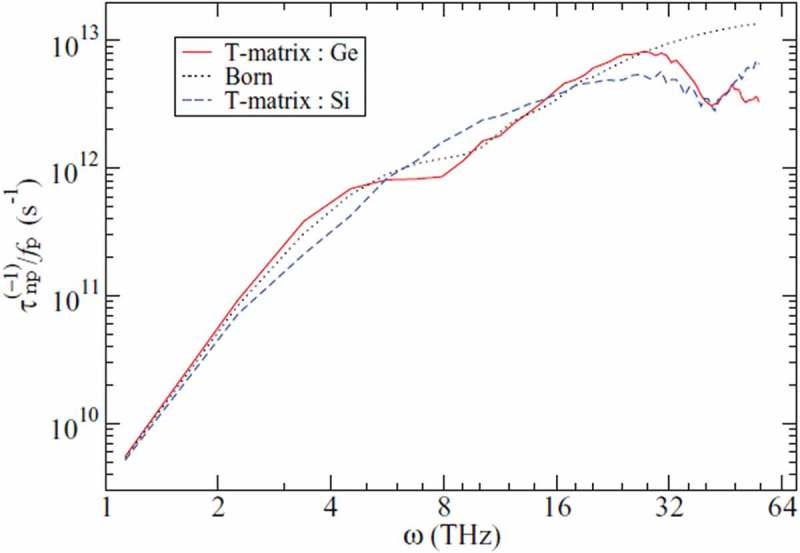
Scattering rate *τ*_np_^−1^ due to Si or Ge nanoparticles embedded in Si_0.5_Ge_0.5_ crystals calculated by the T-matrix method. The rate is normalized by nanoparticle volume fraction *f*_p_ [99]. Reproduced with permission from [99]. Copyright 2011 The American Physical Society.

As introduced above, it is possible to incorporate the alloying effect into the evaluation of phonon transport properties using the Tamura model. However, the force-field difference originated from heterogeneity of the atoms in an alloy crystal is not commonly taken into account in the model. A model utilizing sound speed to incorporate force-field difference has been reported [85] but it lacks in the realistic complexity. Thus the previous studies utilizing ALD have approximated alloys as mass difference system [87–89] even in the case with strong force-field contrast. This is probably the reason why the model fails to reproduce the thermal conductivity of PbTe*_x_*Se_1__−_*_x_* [88], while it works for Si*_x_*Ge_1__−_*_x_* [87] where the interatomic force constants of Si and Ge single crystals are transferable [97]. Another issue is that the validity of the perturbation theory for mass difference is limited to a small mass contrast and low concentration. As the alternative way to overcome these limitations, T-matrix method based on Green’s function method is gaining attention [98–101]. In this method, molecular positions are explicitly taken into account and the effect of higher order scattering due to mass difference can be incorporated, unlike the perturbation model in Equation (4). Figure 3 shows the work by Kundu et al. [99] on the scattering rate due to Si or Ge nanoparticles in Si_0.5_Ge_0.5_ crystals calculated by the T-matrix method. It clearly indicates that the scattering rates caused by heavier and lighter particles are different while they are identical in Tamura model, in other words, the Born approximation (‘Born’ in Figure 3). The study only considered mass difference, but force-field difference can be also incorporated in the T-matrix method. Katre et al. evaluated the effect of B substitution in SiC crystals incorporating local changes in both the masses and interatomic force constants [101]. The study revealed that the scattering rate is three orders of magnitude larger than that calculated accounting for only the mass difference.

**Figure 4.**
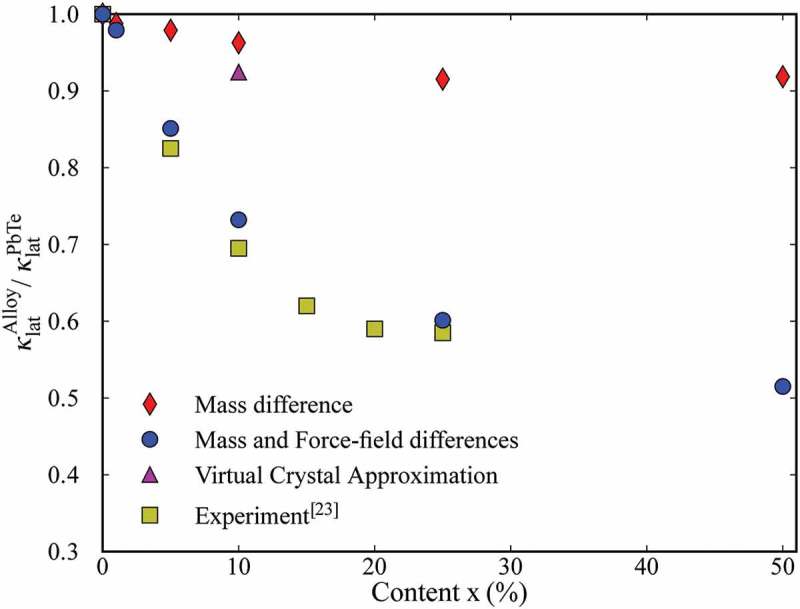
Thermal conductivities of PbTe_1−*x*_Se*_x_* with and without force field difference, normalized by those of pure PbTe crystal. The results are compared with the experiment [109]. Reproduced with permission from [107]. Copyright 2013 IOP publishing.

Another approach to overcome the above limitations of the theoretical models is molecular dynamics (MD) simulation, which solves Newton’s equations of motion with a given intermolecular potential function to obtain the time evolution of the positions and velocities. The method is capable of directly incorporating mass and force-field differences in alloys. In addition, the exact spatial distribution of the atom species in alloys can be incorporated since MD solves the trajectories in real space. Therefore, owing also to its simplicity, MD is widely utilized to evaluate thermal conductivity of alloys [16,102–108]. One of the previous studies [107] performed MD simulations with a potential function obtained from first principles to investigate the effect of force-field difference on the thermal conductivity of PbTe*_x_*Se_1__−_*_x_*. The results have shown that the mass difference alone cannot reproduce the thermal conductivity reduction observed in experiments [109] and the inclusion of the local force field is essential for the reproduction, as shown in Figure 4.

The phonon properties of each mode in alloy can be evaluated by MD using the normal mode decomposition (NMD) method [110–112]. In this method, the time correlations of the decomposed normal modes are recorded. In particular, much attention has been paid to the effect of alloying on relaxation time [113–119]. For example, Baker and Norris reported the influence of long- and short-range order of composition of Si*_x_*Ge_1−*x*_ alloy on the relaxation time, and found that the latter is dominant [116]. Feng et al. validated the Matthiessen’s rule in Equation (3) [117] by exploiting the method. It was found that Equation (3) overestimates the phonon relaxation time and thus thermal conductivity because it does not take into account the coupling between phonon-phonon and phonon-impurity scattering. In this way, MD has been successfully used to investigate not only the total thermal conductivity but also its spectral properties. Note that, while the relaxation time with alloying effect has been gradually unveiled, the variation of group velocity due to alloying has not yet been fully understood [114].

Experiments have also contributed to understanding of alloy effects on phonon transport. Much of it has been realized by the recent development of nanotechnology to fabricate and characterize nanostructures. For instance, strong size effect on the thermal conductivity of Si*_x_*Ge_1__−_*_x_* alloy films was found by time-domain thermoreflectance measurement [120]. Huberman et al. performed transient thermal grating measurements [121] to study the size effect on thermal transport of Si*_x_*Ge_1__−_*_x_* alloy [122]. The strong size dependence of thermal conductivity indicates that the phonons of long MFP, namely low-frequency modes, remains to largely contribute to the thermal conductivity of alloy crystals, which is consistent with the numerical calculations. In addition to the thermal conductivity measurement, the direct probing of phonon dispersion relations and relaxation times of alloys has also been performed by using inelastic x-ray scattering [123]. The observed phonon dispersion relations of PbTe*_x_*Se_1__−_*_x_* alloys have shown good agreement with the results of the calculations under a virtual crystal approximation, which suggests that the approximation is a reasonable to predict the phonon dispersion of the PbTe*_x_*Se_1__−_*_x_* alloys. These experimental approaches are expected to be utilized in various materials and conditions in conjunction with the numerical approaches to further understand the phonon transport of alloys.

## 3. Phonon-boundary scattering in nanocrystals

As introduced in Section 1, various kinds of nanostructured thermoelectric materials have been fabricated in recent years. Among the nanostructures, sintered nanocrystal structures have been shown to be one of the most promising approaches owing to its high performance, relatively low fabrication cost, and high scalability compared with the structures that requires precise fabrication such as superlattices. As for power factor *S*^2^*σ*, it can remain almost unchanged after the sintering [57]. The method is applicable to various materials, not only simple crystals such as Si [124–127] but also the complex alloys and compounds such as Si*_x_*Ge_1__−_*_x_* [128–130], Bi*_x_*Sb_2__−_*_x_*Te_3_ [57,131,132], and half-Heusler [133,134]. The thermal conductivity reduction in the nanostructures originates from phonon scattering at grain boundaries, which reduces the effective MFPs of phonons. Owing to the scattering, effective MFPs of the nanostructures become shorter. Therefore, phonon transmission probabilities (PTPs) at the interfaces are the key properties to understand the thermal conduction in nanocrystals. Here we note that PTP is known to strongly depend on phonon modes. In addition, the geometries of the nanostructures such as the crystal-size distribution affect the thermal conduction. Although the analytical solutions of the effective MFP of phonons in simple structures such as thin films [135–137] or nanowires [138,139] are already well known, they cannot be easily extended to nanocrystals that is much more complex with broad grain size distribution and spatial inhomogeneity. Therefore, to unveil how phonon transport depends on the mode and complex geometry, numerical approaches are beneficial and have been utilized.

The empirical models of frequency-dependent PTP that are widely known and used are the acoustic mismatch model [140] and diffusive mismatch model [141]. However, it is also widely known that these models derived under bold assumptions cannot reproduce thermal boundary resistance observed in experiments. Meanwhile, the numerical approaches such as MD (phonon wave packet simulation) [142], lattice dynamics [143], and atomistic Green’s function [144] have been developed to evaluate the PTP. In particular, atomistic Green’s function approach has become popular and has been applied to analyze various interfaces between heterogeneous structures [144–149] because the frequency-dependent PTP can be obtained with relatively low computational cost. However, these numerical calculations are limited to the case of the simple interfaces, mainly attributed the difficulty in modeling realistic interfaces, namely their geometries and interatomic force fields, rather than the limitation in the computational technique.

**Figure 5.**
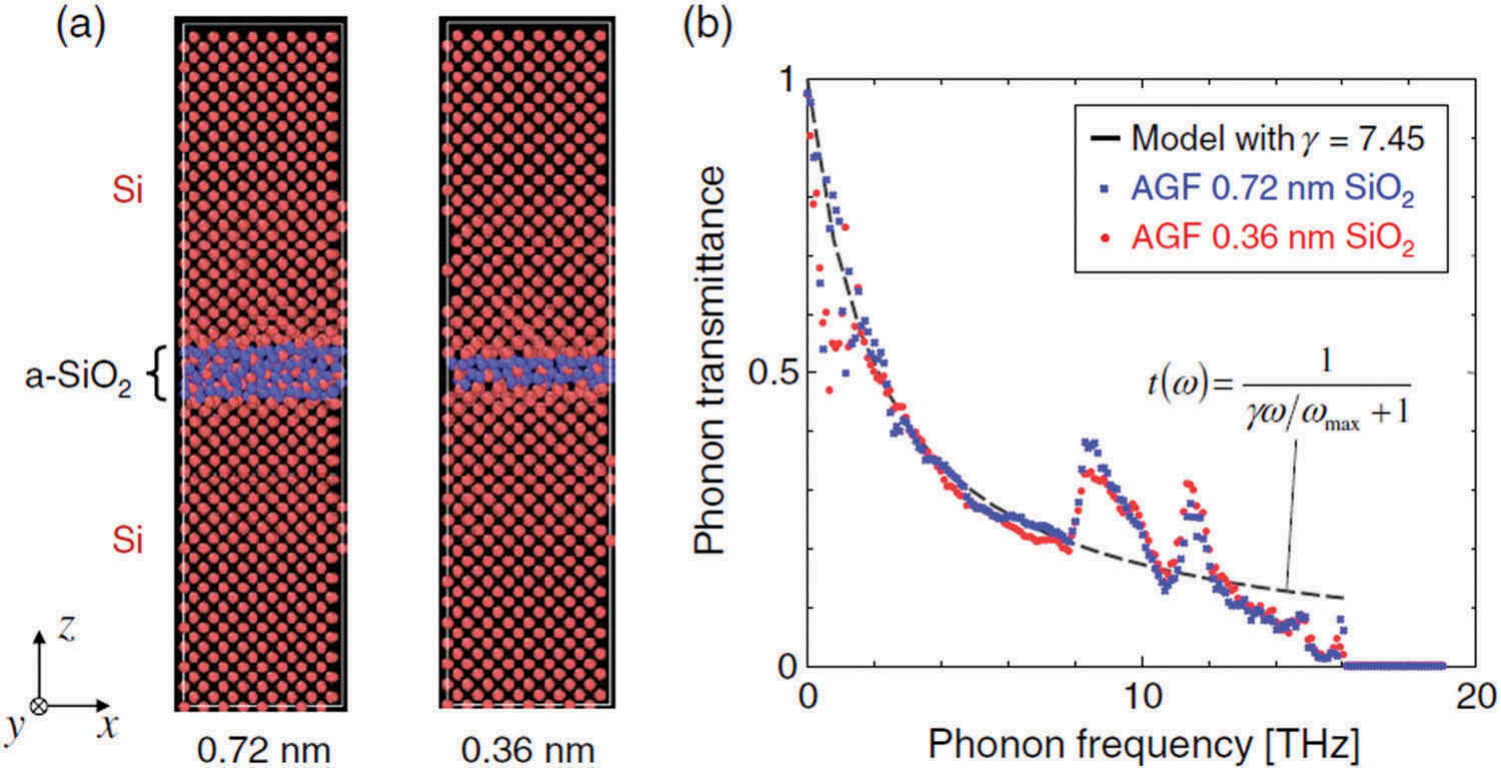
(a) Structure of amorphous SiO_2_ at the interface between crystalline Si layers for the atomistic Green’s function simulations. SiO_2_ layer thicknesses are 0.72 and 0.36 nm. (b) Phonon transmission probabilities (PTPs) across the interfaces. Dashed line represents the fitting curve of Equation (6) [152]. Reproduced with permission from [152]. Copyright 2018 The American Physical Society.

As for the experimental approaches, the PTP can be investigated but still requires inputs from corresponding numerical calculations or theoretical assumptions. For example, PTP at sintered silicon interfaces was investigated based on the thermal boundary conductance obtained by the time-domain thermoreflectance [150]. In the study, the function of PTP *t* is assumed by a simplified equation,

(6) $$t\left(\omega \right)=\frac{1}{\gamma \omega /\omega_{max}+1}$$

which was suggested from the experimentally measured temperature dependence of thermal conductivity of sintered silicon nanocrystals [151]. In Equation (6), *ω*_max_ and *γ* represent the maximum frequency of materials and a fitting parameter. This equation reproduces the frequency dependence of PTP obtained by the previous models and calculations, where it decreases with increasing phonon frequency. Indeed, although the decaying curve in Equation (6) is simplified, a previous study using atomistic Green’s function showed that it agrees with the PTP of silicon interfaces consisting of monolayer amorphous SiO_2_, as shown in Figure 5 [152]. A framework to extract PTP at solid interfaces is also recently reported, where phonon transport properties obtained by ALD with first-principles calculation [14] and time-domain thermoreflectance are utilized [153]. These measurement methods would make contributions to the understanding of the phonon transmission phenomenon at the sintered interface in future.

Several works employed MD simulations to analyze heat-transfer phenomenon in sintered nanocrystals [154–158]. However, the size and number of the nanocrystals are limited due to the computational cost. Therefore, coarse-grained model is needed to analyze thermal conduction in the sintered nanocrystals. A promising way is to solve the Boltzmann transport equation under relaxation time approximations [9],

(7) $$\frac{\partial f_{s}\left(\omega ,r\right)}{\partial t}+v_{s}\left(\omega \right)e\cdot \frac{\partial f_{s}\left(\omega ,r\right)}{\partial r}=-\frac{f_{s}\left(\omega ,r;t\right)-f_{0}\left(\omega ,T\left(r\right)\right)}{\tau_{s}\left(\omega ,T\left(r\right)\right)},$$

which describes phonon transport in terms of nonequilibrium distribution function. Here, *f, f*_0_, **r**, and *T* are the phonon distribution function, Bose-Einstein distribution, position, and temperature, respectively. Equation (7) indicates that time evolution of the distribution of phonons with *ω, s* at **r** is described by the advection and scattering terms. Phonon transport in nanostructures requires characterization of complicated geometries. Monte Carlo (MC) simulation is a good way to resolve such situations. In the method, phonons are treated as particles and their time evolutions of advection and scattering by other phonons and interfaces are explicitly simulated. The frequency dependent phonon properties such as relaxation time and group velocity are required as input parameters. Following the long history of the MC method for Boltzmann transport equation to solve rarefied gas dynamics [159], in recent years, there is a growing number of studies applying the method to phonon transport problems [160–173]. The most notable development of the simulation technique is the variance-reduced MC simulation [165,168,171], which can realize drastically low computational cost by simulating only the deviational particles from a reference distribution.

Other simplified calculation methods have also been developed [174–178]. For example, the test particle method developed in the field of rarefied gas dynamics [179–181] has been applied to the phonon transport [176–178], which is also called the ray-tracing simulation by an analogy with photon. In this method, the transmission probability of particles from one side of an entire structure to the opposite side is calculated. Then the transmission probability gives the effective MFP. The method has been used to investigate the effective MFP of sintered nanocrystals [176]. The nanocrystal geometries were prepared by tuned Voronoi diagrams, where the crystal size *x* with log-normal distribution *P* [Equation (8)] as in realistic polycrystalline materials were numerically generated [151].

(8) $$P\left(x\right)=\frac{1}{\sqrt{2\pi }\sigma x}\exp\left(-\frac{{\left(\ln x\right)}^{2}}{2\sigma^{2}}\right)$$

**Figure 6.**
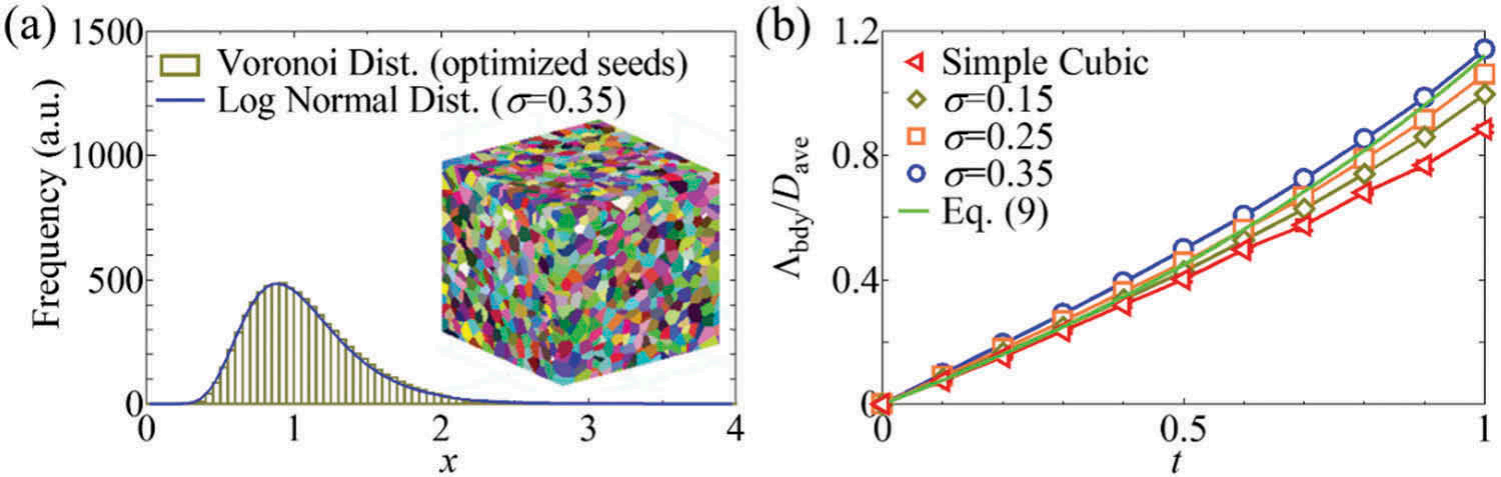
(a) Polycrystalline nanostructures (PNs) and their grain-size distributions generated by Voronoi diagrams using modified seed-point distributions. (b) The phonon transmission probability *t* dependence of effective MFP Λ_bdy_ of simple cubic and realistic crystal size distributions obtained by ray-tracing simulations, and calculated by the simple model in Equation (9) [176]. Reproduced with permission from [176]. Copyright 2015 AIP Publishing.

The descriptor of crystal size distribution *σ* is varied in the range of the experimental results [151] to evaluate its effect on the effective MFP. The constructed crystal structure and the crystal size distribution are shown in Figure 6(a). By performing the simulations, the effective MFP is evaluated as a function of *t* and compared with the simple model that combines MFPs of a superlattice and a square nanowire using the Matthiessen’s rule expressed by Equation (9),

(9) $$\frac{\Lambda_{bdy}}{D_{ave}}={\left[{\left(\frac{3}{4}\frac{t}{1-t}\right)}^{-1}+{\left(1.12\right)}^{-1}\right]}^{-1}$$

where Λ_bdy_ is effective MFP when intrinsic MFP is infinity, *D*_ave_ is average crystal size. The comparison shown in Figure 6(b) indicates that Equation (9) can mostly predict the calculated results. Here we note that the simulation and theory assume the fully diffusive scattering of phonons at the interfaces; thus the effective MFP of high *t* region, or low frequency as seen in Equation (6), can be underestimated. Phonons of extremely long MFP have low frequency and thus are less affected by the interfaces as in Equation (6). Once effective MFP is obtained thermal conductivity can be accessed by utilizing bulk properties of phonons such as group velocity, as in the MC method.

**Figure 7.**
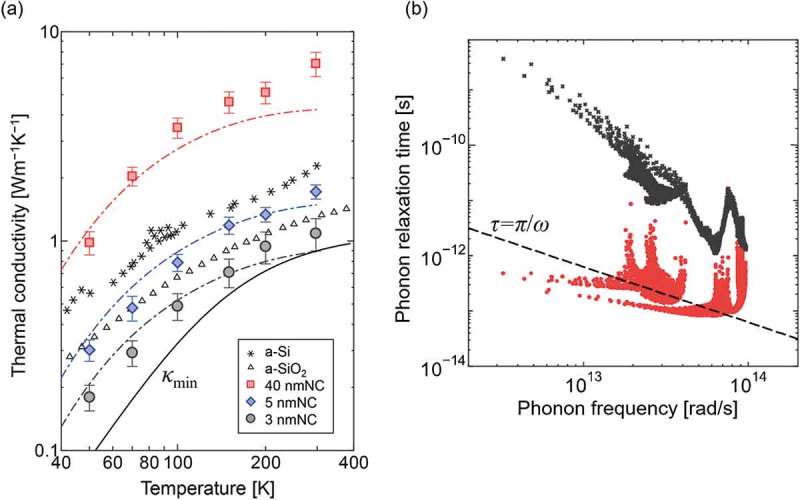
(a) Temperature-dependent thermal conductivity of silicon nanocrystalline (SiNC) structures compared with amorphous Si (asterisks) [183,184] and SiO_2_ (triangles) [185]. Black solid line represents the minimum thermal conductivity model [185]. Dotted lines show thermal conductivity calculated by phonon gas model with inputs from anharmonic lattice dynamics and atomistic Green’s function (AGF) calculations. (b) Frequency-dependent phonon relaxation times of bulk Si (black crosses) and SiNC with grain size of 3 nm (red dots) at 300 K [152]. Reproduced with permission from [152]. Copyright 2018 The American Physical Society.

While it is clear from above that impedance of phonon transport is stronger for smaller grains, an important question now is; what is the limit to suppression of phonon relaxation or mean free path? A widely accepted reference so far is the minimum thermal conductivity scenario, i.e. mean free path of phonon becomes equal to half its wavelength. Aiming to gain further insight into this aspect, measurements have been performed for nanocrystal grains with diameters of 3, 5, and 40 nm [182]. The samples are epitaxial silicon nanocrystalline (SiNC) structures composed of grains separated by ultrathin silicon-oxide films. The ultrathin silicon-oxide films have nanowindows that connect the nanograins preserving the crystal orientation, and thus, allowing electrons to travel with coherent wavefunctions. Thermal conductivities of SiNC structures are significantly below the values of bulk amorphous Si and SiO_2_. To identify the mechanism of the low thermal conductivity, its temperature dependence was measured. Figure 7(a) summarizes the temperature dependence of the thermal conductivity of SiNC structures compared with those of amorphous Si and amorphous SiO_2_ [183–185]. The temperature dependence was successfully reproduced by a phonon gas kinetics model with intrinsic (bulk) transport properties obtained by first-principle-based anharmonic lattice and phonon transmittance across silicon-oxide films obtained by atomistic Green’s function. Consequently, as shown in Figure 7(b), the analysis revealed that relaxation time of acoustic phonons in the case of 3-nm SiNC structures are suppressed below the minimum thermal conductivity scenario.

When applying the above MC simulations, ray-tracing calculations, or theoretical model on the basis of phonon gas kinetics, a relevant question is the applicability to utilize the bulk properties to predict thermal conduction in nanostructures, where the phonon modes may be altered by confinement effect. This validity has not yet been rigorously proven, however in addition to the successful reproduction of SiNC thermal conductivity with the phonon gas kinetics model using bulk phonon properties, molecular dynamics simulations have shown that the partial density of states of Si nanocrystals with a diameter of 3 nm is similar to that of bulk Si [152]. Therefore, reported studies suggest that the phonon kinetic model with bulk phonon transport inside the grain and PTP at the interference is relevant for nanostructures of broad length scales, and thus, the multiscale phonon approach introduced here is useful for simulating complex geometries in practical materials such as nanocrystals.

## 4. Resonance effect by embedded nanoparticles

Embedded nanoparticles, as well as the sintered nanocrystals, have been shown to enhance performance of thermoelectric materials. The merits of the structures are that coherent interfaces and small band offsets between host and embedded materials can be realized by suitable selection of nanoparticle. Such interfaces allow carrier transmission [186], unlike the sintered interfaces that inevitably result in degraded electrical conductivity. Yet, thermal conductivity can be significantly decreased as the phonons are scattered by the particles. The scattering is often described by the combination of impurity and geometrical scattering [187,188]. Previous works have identified the correlation between the size distribution of the nanoparticles and thermal conductivity, and investigated the optimized distribution that can effectively scatter phonons with a broad range of MFPs [189]. An experimental study has also shown that the combination of the nanoparticles, impurities, and boundary scattering of phonons is effective in reducing thermal conductivity because the multiscale structures hierarchically scatter phonons with a range of MFPs [63].

**Figure 8.**
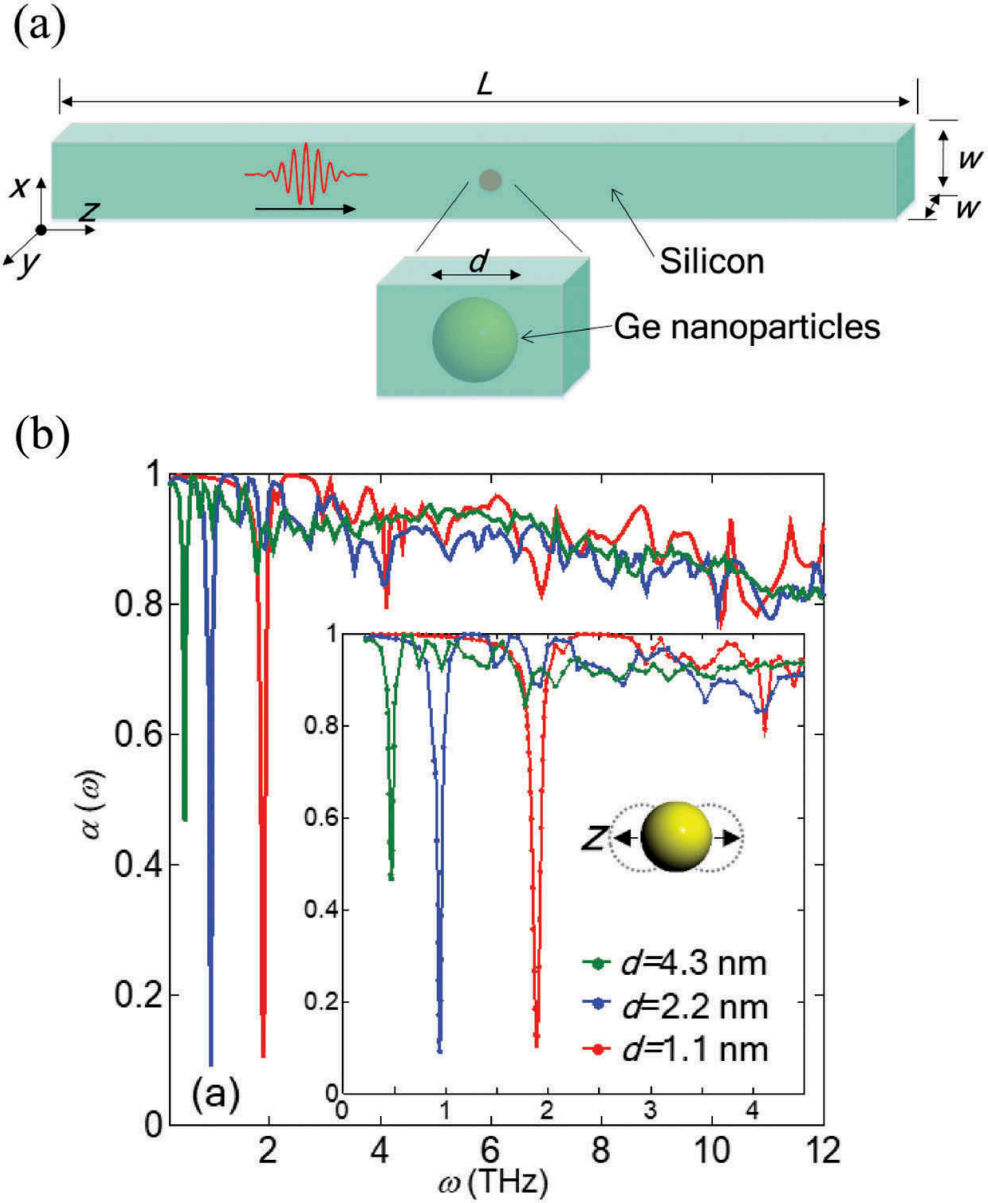
(a) Schematic diagram of the system where the Ge nanoparticles are embedded in a Si matrix. (b) Frequency-dependent transmission probability α(*ω*) of longitudinal acoustic phonon calculated by MD simulations. Inset diagram shows the motion of the particle in the rattling mode [192]. Reproduced with permission from [192]. Copyright 2017 The American Physical Society.

Molecular dynamics simulations have also been conducted to understand the phonon scattering with nanoparticles [190–192]. It was shown that these nanoparticles can act as resonators that impedes phonon transport [192]. Note that the phonon resonance effect and its influence on thermal conductivity has also been investigated in substructures such as thin films with surface pillars [193–198]. Figure 8 shows that a schematic of wave packet simulation in silicon matrix with embedded germanium nanoparticles, and the PTP through the system. As can be seen in Figure 8, sharp and significant transmission-probability dips are observed at resonant frequencies. The study also revealed that the dips can be made more pronounced by arraying multiple nanoparticles. In addition, the frequency of the dips can be tuned through the distance between arraying particles. A similar result was reported in a previous work where the influence of the distance between two impurity atoms on the phonon scattering rate was investigated by the T-matrix method [100].

Detail discussion of the mechanism of various resonance effects can be learned also from the series of modeling works on atomistically controlled structures [199–201]. Han et al. employed MD wave packet simulations and revealed that a single crystal plane of heterogeneous atom arrays embedded in a host crystal can strongly impede phonons of a specific frequency [199]. The mechanism originates from a wave interference, namely antiresonance [202], where the phonons coming by the paths through the impurity atom bonds and host atom bonds interfere destructively.

Blocking the phonon transport through resonance effect originated from the nanoparticles is beneficial for suppressing the thermal conductivity of specific phonon modes. For example, when some of the phonon modes still significantly contribute to thermal conductivity even after alloying and nanostructuring, the tuned resonance effect leads to a further reduction of thermal conductivity. More importantly, it can significantly block the lower frequency range of phonon transport, which is hardly terminated by other nanostructures. Here the problem is how to realize the optimized arrangement of nanoparticles in actual materials. However, when the technical problem is resolved in near future, ultimately low thermal conductivity materials may be realized by the phonon engineering.

## 5. Summary and perspectives

We reviewed the recent theoretical, numerical, and experimental works on effects of nanostructure on phonon transport spectrum, whose progress can lead to design of superior thermoelectric materials. There has been a strong progress in theoretical and numerical methods to analyze alloy scattering, boundary scattering, and structure-induced interference and resonance of phonon transport. Together with mesoscopic approach that lumps multiple effects and quantifies the overall impact on thermal conductivity, it has become possible to design a structure that impedes phonon transport in the entire spectral domain with noticeable contribution to thermal conductivity, ranging in general from subterahertz to tens of terahertz. As many interesting structures have been already fabricated experimentally and have been shown to enhance *ZT* by reducing thermal conductivity, such designability provided by theoretical and numerical calculations is expected to contribute to further enhancement in terms of controlling spectral (mode-dependent) thermal conductivity. There, machine learning can be a useful tool to realize optimal design [203–205].

## References

[1] Goldsmid HJ. Introduction to thermoelectricity. Heidelberg, New York: Springer; 2010 (Springer series in materials science; 121).

[2] Rowe DM. CRC handbook of thermoelectrics. Boca Raton, FL: CRC Press; 1995.

[3] Akinaga H, Fujita H, Mizuguchi M, et al Focus on advanced materials for energy harvesting: prospects and approaches of energy harvesting technologies. Sci Technol Adv Mater. 2018;19(1):543–544. 30108664 10.1080/14686996.2018.1491165 PMC6084487

[4] Pei YZ, Shi XY, LaLonde A, et al Convergence of electronic bands for high performance bulk thermoelectrics. Nature. 2011;473(7345):66–69. 21544143 10.1038/nature09996

[5] Vashaee D, Shakouri A Improved thermoelectric power factor in metal-based superlattices. Phys Rev Lett. 2004;92(10):106103. 15089220 10.1103/PhysRevLett.92.106103

[6] Zide JMO, Vashaee D, Bian ZX, et al Demonstration of electron filtering to increase the Seebeck coefficient in In_0.53_Ga_0.47_As/In_0.53_Ga_0.28_Al_0.19_As superlattices. Phys Rev B. 2006;74:20.

[7] Heremans JP, Jovovic V, Toberer ES, et al Enhancement of thermoelectric efficiency in PbTe by distortion of the electronic density of states. Science. 2008;321(5888):554–557. 18653890 10.1126/science.1159725

[8] Zhang QY, Wang H, Liu WS, et al Enhancement of thermoelectric figure-of-merit by resonant states of aluminium doping in lead selenide. Energy Environ Sci. 2012;5(1):5246–5251.

[9] Srivastava GP The physics of phonons. Bristol, Philadelphia: A. Hilger; 1990.

[10] Debye P The theory of specific warmth. Ann Phys Berlin. 1912;39(14):789–839.

[11] Rowe DM Thermoelectrics handbook: macro to nano. Boca Raton: CRC/Taylor & Francis; 2006.

[12] Yang F, Dames C Mean free path spectra as a tool to understand thermal conductivity in bulk and nanostructures. Phys Rev B. 2013;87(3):035437.

[13] Aketo D, Shiga T, Shiomi J Scaling laws of cumulative thermal conductivity for short and long phonon mean free paths. Appl Phys Lett. 2014;105:13.

[14] Broido DA, Malorny M, Birner G, et al Intrinsic lattice thermal conductivity of semiconductors from first principles. Appl Phys Lett. 2007;91(23):231922.

[15] Esfarjani K, Chen G, Stokes HT Heat transport in silicon from first-principles calculations. Phys Rev B. 2011;84(8):085204.

[16] Shiomi J, Esfarjani K, Chen G Thermal conductivity of half-Heusler compounds from first-principles calculations. Phys Rev B. 2011;84(10):104302.

[17] Shiga T, Shiomi J, Ma J, et al Microscopic mechanism of low thermal conductivity in lead telluride. Phys Rev B. 2012;85(15):155203.

[18] Tadano T, Gohda Y, Tsuneyuki S Anharmonic force constants extracted from first-principles molecular dynamics: applications to heat transfer simulations. J Phys Condens Matter. 2014;26(22):225402. 24824156 10.1088/0953-8984/26/22/225402

[19] Delaire O, Ma J, Marty K, et al Giant anharmonic phonon scattering in PbTe. Nat Mater. 2011;10(8):614–619. 21642983 10.1038/nmat3035

[20] Ma J, Delaire O, May AF, et al Glass-like phonon scattering from a spontaneous nanostructure in AgSbTe_2_. Nat Nanotechnol. 2013;8(6):445–451. 23728075 10.1038/nnano.2013.95

[21] Burkel E, Peisl J, Dorner B Observation of inelastic X-ray-scattering from phonons. Europhys Lett. 1987;3(8):957–961.

[22] Uchiyama H, Oshima Y, Patterson R, et al Phonon lifetime observation in epitaxial ScN film with inelastic X-ray scattering spectroscopy. Phys Rev Lett. 2018;120(23):235901. 29932681 10.1103/PhysRevLett.120.235901

[23] Koh YK, Cahill DG Frequency dependence of the thermal conductivity of semiconductor alloys. Phys Rev B. 2007;76(7):075207.

[24] Minnich AJ, Johnson JA, Schmidt AJ, et al Thermal conductivity spectroscopy technique to measure phonon mean free paths. Phys Rev Lett. 2011;107(9):095901. 21929254 10.1103/PhysRevLett.107.095901

[25] Regner KT, Sellan DP, Su Z, et al Broadband phonon mean free path contributions to thermal conductivity measured using frequency domain thermoreflectance. Nat Commun. 2013;4:1640. 23535661 10.1038/ncomms2630

[26] Hu YJ, Zeng LP, Minnich AJ, et al Spectral mapping of thermal conductivity through nanoscale ballistic transport. Nat Nanotechnol. 2015;10(8):701–706. 26030656 10.1038/nnano.2015.109

[27] Steele MC, Rosi FD. Thermal conductivity and thermoelectric power of germanium-silicon alloys. J Appl Phys. 1958;29(11):1517–1520.

[28] Dismukes JP, Ekstrom E, Beers DS, et al Thermal + electrical properties of heavily doped Ge-Si alloys up to 1300 degrees K. J Appl Phys. 1964;35(10):2899-&.

[29] Vining CB, Laskow W, Hanson JO, et al Thermoelectric properties of pressure-sintered Si_0.8_Ge_0.2_ thermoelectric alloys. J Appl Phys. 1991;69(8):4333–4340.

[30] Testardi LR, Bierly JN, Donahoe FJ Transport properties of p-type Bi_2_Te_3_ Sb_2_Te_3_ alloys in the temperature range 80–370[deg] K. J Phys Chem Solids. 1962;23(9):1209–1217.

[31] Yim WM, Rosi FD. Compound tellurides and their alloys for peltier cooling – review. Solid State Electron. 1972;15(10):1121–1140.

[32] Caillat T, Carle M, Pierrat P, et al Thermoelectric properties of (Bi_x_sb_1-X_)_2_Te_3_ single-crystal solid-solutions grown by the thm method. J Phys Chem Solids. 1992;53(8):1121–1129.

[33] Kim HS, Heinz NA, Gibbs ZM, et al High thermoelectric performance in (Bi_0.25_Sb_0.75_)_2_Te_3_ due to band convergence and improved by carrier concentration control. Mater Today. 2017;20(8):452–459.

[34] Zaitsev VK, Fedorov MI, Gurieva EA, et al Highly effective Mg_2_Si_1−x_Sn_x_ thermoelectrics. Phys Rev B. 2006;74(4):045207.

[35] Zhang Q, Yin H, Zhao XB, et al Thermoelectric properties of n-type Mg_2_Si_0.6-y_Sb_y_Sn_0.4_ compounds. Phys Status Solidi. 2008;205(7):1657–1661.

[36] Liu W, Tan X, Yin K, et al Convergence of conduction bands as a means of enhancing thermoelectric performance of n-type Mg_2_Si_1-x_Sn_x_ solid solutions. Phys Rev Lett. 2012;108(16):166601. 22680741 10.1103/PhysRevLett.108.166601

[37] Zhang Q, Cheng L, Liu W, et al Low effective mass and carrier concentration optimization for high performance p-type Mg_2(1-x)_ Li_2x_Si_0.3_Sn_0.7_ solid solutions. Phys Chem Chem Phys. 2014;16(43):23576–23583. 25178356 10.1039/c4cp03468f

[38] Smith GE, Wolfe R Thermoelectric properties of bismuth-antimony alloys. J Appl Phys. 1962;33(3):841–846.

[39] Poudeu PFP, D’Angelo J, Kong HJ, et al Nanostructures versus solid solutions: low lattice thermal conductivity and enhanced thermoelectric figure of merit in Pb_9.6_Sb_0.2_Te_10-x_Se_x_ bulk materials. J Am Chem Soc. 2006;128(44):14347–14355. 17076508 10.1021/ja0647811

[40] Zhang Q, Cao F, Liu WS, et al Heavy doping and band engineering by potassium to improve the thermoelectric figure of merit in p-Type PbTe, PbSe, and PbTe_1-y_Se_y_. J Am Chem Soc. 2012;134(24):10031–10038. 22676702 10.1021/ja301245b

[41] Uher C, Yang J, Hu S, et al Transport properties of pure and doped MNiSn (M=Zr, Hf). Phys Rev B. 1999;59(13):8615–8621.

[42] Bhattacharya S, Pope AL, Littleton RT, et al Effect of Sb doping on the thermoelectric properties of Ti-based half-Heusler compounds, TiNiSn_1-x_Sb_x_. Appl Phys Lett. 2000;77(16):2476–2478.

[43] Xia Y, Bhattacharya S, Ponnambalam V, et al Thermoelectric properties of semimetallic (Zr,Hf)CoSb half-Heusler phases. J Appl Phys. 2000;88(4):1952–1955.

[44] Yang J, Meisner GP, Chen L Strain field fluctuation effects on lattice thermal conductivity of ZrNiSn-based thermoelectric compounds. Appl Phys Lett. 2004;85(7):1140–1142.

[45] Sakurada S, Shutoh N Effect of Ti substitution on the thermoelectric properties of (Zr,Hf)NiSn half-Heusler compounds. Appl Phys Lett. 2005;86:8.

[46] Sekimoto T, Kurosaki K, Muta H, et al High-thermoelectric figure of merit realized in p-type half-Heusler compounds: ZrCoSn_x_Sb_1-x_. Jpn J Appl Phys. 2007;46(25–28):L673–L675.

[47] Majumdar A Thermoelectricity in semiconductor nanostructures. Science. 2004;303(5659):777–778. 14764859 10.1126/science.1093164

[48] Hicks LD, Dresselhaus MS Effect of quantum-well structures on the thermoelectric figure of merit. Phys Rev B. 1993;47(19):12727–12731. 10.1103/physrevb.47.12727 10005469

[49] Hicks LD, Dresselhaus MS Thermoelectric figure of merit of a one-dimensional conductor. Phys Rev B. 1993;47(24):16631–16634. 10.1103/physrevb.47.16631 10006109

[50] Mahan GD, Sofo JO The best thermoelectric. Proc Natl Acad Sci USA. 1996;93(15):7436–7439. 11607692 10.1073/pnas.93.15.7436 PMC38761

[51] Casimir HBG Note on the conduction of heat in crystals. Physica. 1938;5:495–500.

[52] Venkatasubramanian R, Siivola E, Colpitts T, et al Thin-film thermoelectric devices with high room-temperature figures of merit. Nature. 2001;413(6856):597–602. 11595940 10.1038/35098012

[53] Harman TC, Taylor PJ, Walsh MP, et al Quantum dot superlattice thermoelectric materials and devices. Science. 2002;297(5590):2229–2232. 12351781 10.1126/science.1072886

[54] Tian Z, Lee S, Chen G. Heat transfer in thermoelectric materials and devices. J Heat Transfer. 2013;135(6):061605.

[55] Hochbaum AI, Chen R, Delgado RD, et al Enhanced thermoelectric performance of rough silicon nanowires. Nature. 2008;451(7175):163–167. 18185582 10.1038/nature06381

[56] Boukai AI, Bunimovich Y, Tahir-Kheli J, et al Silicon nanowires as efficient thermoelectric materials. Nature. 2008;451(7175):168–171. 18185583 10.1038/nature06458

[57] Poudel B, Hao Q, Ma Y, et al High-thermoelectric performance of nanostructured bismuth antimony telluride bulk alloys. Science. 2008;320(5876):634–638. 18356488 10.1126/science.1156446

[58] Il Kim S, Lee KH, Mun HA, et al Dense dislocation arrays embedded in grain boundaries for high-performance bulk thermoelectrics. Science. 2015;348(6230):109–114. 25838382 10.1126/science.aaa4166

[59] Tang J, Wang HT, Lee DH, et al Holey silicon as an efficient thermoelectric material. Nano Lett. 2010;10(10):4279–4283. 20839780 10.1021/nl102931z

[60] Kashiwagi M, Hirata S, Harada K, et al Enhanced figure of merit of a porous thin film of bismuth antimony telluride. Appl Phys Lett. 2011;98:2.

[61] Hsu KF, Loo S, Guo F, et al Cubic AgPb_m_SbTe_2+m_: bulk thermoelectric materials with high figure of merit. Science. 2004;303(5659):818–821. 14764873 10.1126/science.1092963

[62] Biswas K, He JQ, Zhang QC, et al Strained endotaxial nanostructures with high thermoelectric figure of merit. Nat Chem. 2011;3(2):160–166. 21258390 10.1038/nchem.955

[63] Biswas K, He J, Blum ID, et al High-performance bulk thermoelectrics with all-scale hierarchical architectures. Nature. 2012;489(7416):414–418. 22996556 10.1038/nature11439

[64] Miura A, Kikkawa T, Iguchi R, et al Probing length-scale separation of thermal and spin currents by nanostructuring YIG. Phys Rev Mater. 2017;1(1):014601.

[65] Cahill DG, Ford WK, Goodson KE, et al Nanoscale thermal transport. J Appl Phy. 2003;93(2):793–818.

[66] Snyder GJ, Toberer ES Complex thermoelectric materials. Nat Mater. 2008;7(2):105–114. 18219332 10.1038/nmat2090

[67] Minnich AJ, Dresselhaus MS, Ren ZF, et al Bulk nanostructured thermoelectric materials: current research and future prospects. Energy Environ Sci. 2009;2(5):466.

[68] Kanatzidis MG Nanostructured thermoelectrics: the new paradigm?. Chem Mater. 2010;22(3):648–659.

[69] Luo T, Chen G Nanoscale heat transfer–from computation to experiment. Phys Chem Chem Phys. 2013;15(10):3389–3412. 23361372 10.1039/c2cp43771f

[70] Cahill DG, Braun PV, Chen G, et al Nanoscale thermal transport. II. 2003–2012. Appl Phys Rev. 2014;1(1):011305.

[71] Minnich AJ Advances in the measurement and computation of thermal phonon transport properties. J Phys Condens Mat. 2015;27:5. 10.1088/0953-8984/27/5/053202 25603881

[72] Zeier WG, Zevalkink A, Gibbs ZM, et al Thinking like a chemist: intuition in thermoelectric materials. Angew Chem Int Ed. 2016;55(24):6826–6841. 10.1002/anie.201508381 27111867

[73] Shiomi J Research update: phonon engineering of nanocrystalline silicon thermoelectrics. APL Mater. 2016;4:10.

[74] Tan GJ, Zhao LD, Kanatzidis MG Rationally designing high-performance bulk thermoelectric materials. Chem Rev. 2016;116(19):12123–12149. 27580481 10.1021/acs.chemrev.6b00255

[75] Volz S, Shiomi J, Nomura M, et al Heat conduction in nanostructured materials. J Therm Sci Tech. 2016;11:1.

[76] Nozariasbmarz A, Agarwal A, Coutant ZA, et al Thermoelectric silicides: a review. Jpn J Appl Phys. 2017;56:5.

[77] Mori T Novel principles and nanostructuring methods for enhanced thermoelectrics. Small. 2017;13:45. 10.1002/smll.201702013 28961360

[78] Nakamura Y Nanostructure design for drastic reduction of thermal conductivity while preserving high electrical conductivity. Sci Technol Adv Mater. 2018;19(1):31–43. 29371907 10.1080/14686996.2017.1413918 PMC5769778

[79] Chen ZW, Zhang XY, Pei YZ Manipulation of phonon transport in thermoelectrics. Adv Mater. 2018;30:17. 10.1002/adma.201705617 29399915

[80] Liu ZH, Mao J, Liu TH, et al Nano-microstructural control of phonon engineering for thermoelectric energy harvesting. MRS Bull. 2018;43(3):181–186.

[81] Liang Z, Hu M. Tutorial: determination of thermal boundary resistance by molecular dynamics simulations. J Appl Phys. 2018;123:19.

[82] Nomura M, Shiomi J, Shiga T, et al Thermal phonon engineering by tailored nanostructures. Jpn J Appl Phys. 2018;57:8.

[83] Anufrievi M, Nomura M. Phonon and heat transport control using pillar based phononic crystals. Sci Technol Adv Mater. 2018;19(1):863–870. 10.1080/14686996.2018.1542524 PMC6249554 30479674

[84] Ashcroft NW, Mermin ND Solid state physics. New York: Holt; 1976.

[85] Klemens PG The scattering of low-frequency lattice waves by static imperfections. Proc Phys Soc Lond A. 1955;68(12):1113–1128.

[86] Tamura S-I Isotope scattering of dispersive phonons in Ge. Phys Rev B. 1983;27(2):858–866.

[87] Garg J, Bonini N, Kozinsky B, et al Role of disorder and anharmonicity in the thermal conductivity of silicon-germanium alloys: a first-principles study. Phys Rev Lett. 2011;106(4):045901. 21405336 10.1103/PhysRevLett.106.045901

[88] Tian Z, Garg J, Esfarjani K, et al Phonon conduction in PbSe, PbTe, and PbTe_1−x_Se_x_ from first-principles calculations. Phys Rev B. 2012;85(18):184303.

[89] Lee S, Esfarjani K, Mendoza J, et al Lattice thermal conductivity of Bi, Sb, and Bi-Sb alloy from first principles. Phys Rev B. 2014;89(8):085206.

[90] Li W, Lindsay L, Broido DA, et al Thermal conductivity of bulk and nanowire Mg_2_Si_x_Sn_1−x_ alloys from first principles. Phys Rev B. 2012;86(17):174307.

[91] Eliassen SNH, Katre A, Madsen GKH, et al Lattice thermal conductivity of Ti_x_Zr_y_Hf_1-x-y_NiSn half-Heusler alloys calculated from first principles: key role of nature of phonon modes. Phys Rev B. 2017;95(4):045202.

[92] Xia Y, Hodges JM, Kanatzidis MG, et al Lattice thermal transport in group II-alloyed PbTe. Appl Phys Lett. 2018;112:18.

[93] Murphy RM, Murray ÉD, Fahy S, et al Ferroelectric phase transition and the lattice thermal conductivity of Pb_1-x_Ge_x_Te alloys. Phys Rev B. 2017;95(14):144302.

[94] Abeles B Lattice thermal conductivity of disordered semiconductor alloys at high temperatures. Phys Rev. 1963;131(5):1906–1911.

[95] Lindsay L, Broido DA, Reinecke TL *Ab initio* thermal transport in compound semiconductors. Phys Rev B. 2013;87(16):165201.

[96] Togo A, Chaput L, Tanaka I Distributions of phonon lifetimes in Brillouin zones. Phys Rev B. 2015;91(9):094306.

[97] Murakami T, Hori T, Shiga T, et al Probing and tuning inelastic phonon conductance across finite-thickness interface. Appl Phys Express. 2014;7(12):121801.

[98] Mingo N, Esfarjani K, Broido DA, et al Cluster scattering effects on phonon conduction in graphene. Phys Rev B. 2010;81(4):045408.

[99] Kundu A, Mingo N, Broido DA, et al Role of light and heavy embedded nanoparticles on the thermal conductivity of SiGe alloys. Phys Rev B. 2011;84(12):125426.

[100] Mendoza J, Esfarjani K, Chen G An ab initio study of multiple phonon scattering resonances in silicon germanium alloys. J Appl Phys. 2015;117:17.

[101] Katre A, Carrete J, Dongre B, et al Exceptionally strong phonon scattering by B substitution in cubic SiC. Phys Rev Lett. 2017;119(7):075902. 28949692 10.1103/PhysRevLett.119.075902

[102] Skye A, Schelling PK Thermal resistivity of Si–Ge alloys by molecular-dynamics simulation. J Appl Phys. 2008;103(11):113524.

[103] Chen J, Zhang G, Li BW Tunable thermal conductivity of Si_1-x_Ge_x_ nanowires. Appl Phys Lett. 2009;95:7.

[104] Landry ES, McGaughey AJH Effect of interfacial species mixing on phonon transport in semiconductor superlattices. Phys Rev B. 2009;79(7):075316.

[105] He Y, Savic I, Donadio D, et al Lattice thermal conductivity of semiconducting bulk materials: atomistic simulations. Phys Chem Chem Phys. 2012;14(47):16209–16222. 23060011 10.1039/c2cp42394d

[106] Duda JC, English TS, Jordan DA, et al Controlling thermal conductivity of alloys via atomic ordering. J Heat Trans. 2012;134:1.

[107] Murakami T, Shiga T, Hori T, et al Importance of local force fields on lattice thermal conductivity reduction in PbTe_1−x_Se_x_ alloys. EPL (Europhysics Letters). 2013;102(4):46002.

[108] Kandemir A, Ozden A, Cagin T, et al Thermal conductivity engineering of bulk and one-dimensional Si-Ge nanoarchitectures. Sci Technol Adv Mater. 2017;18(1):187–196. 28469733 10.1080/14686996.2017.1288065 PMC5404179

[109] Wang H, LaLonde AD, Pei YZ, et al The criteria for beneficial disorder in thermoelectric solid solutions. Adv Funct Mater. 2013;23(12):1586–1596.

[110] Ladd AJC, Moran B, Hoover WG Lattice thermal conductivity: A comparison of molecular dynamics and anharmonic lattice dynamics. Phys Rev B. 1986;34(8):5058–5064. 10.1103/physrevb.34.5058 9940328

[111] McGaughey AJH, Kaviany M Quantitative validation of the Boltzmann transport equation phonon thermal conductivity model under the single-mode relaxation time approximation. Phys Rev B. 2004;69(9):094303.

[112] Larkin JM, Turney JE, Massicotte AD, et al Comparison and evaluation of spectral energy methods for predicting phonon properties. J Comput Theor Nanosci. 2014;11(1):249–256.

[113] Hori T, Shiga T, Shiomi J Phonon transport analysis of silicon germanium alloys using molecular dynamics simulations. J Appl Phys. 2013;113(20):203514.

[114] Larkin JM, McGaughey AJH Predicting alloy vibrational mode properties using lattice dynamics calculations, molecular dynamics simulations, and the virtual crystal approximation. J Appl Phys. 2013;114(2):023507.

[115] Shiga T, Hori T, Shiomi J Influence of mass contrast in alloy phonon scattering. Jpn J Appl Phys. 2014;53:2.

[116] Baker CH, Norris PM Effect of long- and short-range order on SiGe alloy thermal conductivity: molecular dynamics simulation. Phys Rev B. 2015;91(18):180302.

[117] Feng T, Qiu B, Ruan X Coupling between phonon-phonon and phonon-impurity scattering: A critical revisit of the spectral Matthiessen’s rule. Phys Rev B. 2015;92(23):235206.

[118] Lee Y, Pak AJ, Hwang GS What is the thermal conductivity limit of silicon germanium alloys? Phys Chem Chem Phys. 2016;18(29):19544–19548. 27398924 10.1039/c6cp04388g

[119] Nie JH, Ranganathan R, Liang Z, et al Structural vs. compositional disorder in thermal conductivity reduction of SiGe alloys. J Appl Phys. 2017;122(4).

[120] Cheaito R, Duda JC, Beechem TE, et al Experimental investigation of size effects on the thermal conductivity of Silicon-Germanium alloy thin films. Phys Rev Lett. 2012;109(19):195901. 23215405 10.1103/PhysRevLett.109.195901

[121] Johnson JA, Maznev AA, Cuffe J, et al Direct measurement of room-temperature nondiffusive thermal transport over micron distances in a silicon membrane. Phys Rev Lett. 2013;110(2). 10.1103/PhysRevLett.110.025901 23383915

[122] Huberman S, Chiloyan V, Duncan RA, et al Unifying first-principles theoretical predictions and experimental measurements of size effects in thermal transport in SiGe alloys. Phys Rev Mater. 2017;1(5):054601.

[123] Tian ZT, Li MD, Ren ZS, et al Inelastic x-ray scattering measurements of phonon dispersion and lifetimes in PbTe_1-x_Se_x_ alloys. J Phys Condens Mat. 2015;27:37. 10.1088/0953-8984/27/37/375403 26328745

[124] Bux SK, Blair RG, Gogna PK, et al Nanostructured Bulk Silicon as an effective thermoelectric material. Adv Funct Mater. 2009;19(15):2445–2452.

[125] Yusufu A, Kurosaki K, Miyazaki Y, et al Bottom-up nanostructured bulk silicon: a practical high-efficiency thermoelectric material. Nanoscale. 2014;6(22):13921–13927. 25311105 10.1039/c4nr04470c

[126] Claudio T, Stein N, Stroppa DG, et al Nanocrystalline silicon: lattice dynamics and enhanced thermoelectric properties. Phys Chem Chem Phys. 2014;16(47):25701–25709. 24848359 10.1039/c3cp53749h

[127] Miura A, Zhou S, Nozaki T, et al Crystalline-amorphous silicon nanocomposites with reduced thermal conductivity for bulk thermoelectrics. ACS Appl Mater Inter. 2015;7(24):13484–13489. 10.1021/acsami.5b02537 26046688

[128] Wang XW, Lee H, Lan YC, et al Enhanced thermoelectric figure of merit in nanostructured n-type silicon germanium bulk alloy. Appl Phys Lett. 2008;93(19):193121. 10.1021/nl8026795 19367858

[129] Joshi G, Lee H, Lan YC, et al Enhanced thermoelectric figure-of-merit in nanostructured p-type silicon germanium bulk alloys. Nano Lett. 2008;8(12):4670–4674. 19367858 10.1021/nl8026795

[130] Zhu GH, Lee H, Lan YC, et al Increased phonon scattering by nanograins and point defects in nanostructured silicon with a low concentration of germanium. Phys Rev Lett. 2009;102(19):196803. 19518985 10.1103/PhysRevLett.102.196803

[131] Ma Y, Hao Q, Poudel B, et al Enhanced thermoelectric figure-of-merit in p-type nanostructured bismuth antimony tellurium alloys made from elemental chunks. Nano Lett. 2008;8(8):2580–2584. 18624384 10.1021/nl8009928

[132] Lan YC, Poudel B, Ma Y, et al Structure study of bulk nanograined thermoelectric bismuth antimony telluride. Nano Lett. 2009;9(4):1419–1422. 19243189 10.1021/nl803235n

[133] Yan X, Joshi G, Liu W, et al Enhanced thermoelectric figure of merit of p-type half-Heuslers. Nano Lett. 2011;11(2):556–560. 21186782 10.1021/nl104138t

[134] Joshi G, Dahal T, Chen S, et al Enhancement of thermoelectric figure-of-merit at low temperatures by titanium substitution for hafnium in n-type half-Heuslers Hf_0.75−x_Ti_x_Zr_0.25_NiSn_0.99_Sb_0.01_. Nano Energy. 2013;2(1):82–87.

[135] Fuchs K The conductivity of thin metallic films according to the electron theory of metals. Proc Camb Philos Soc. 1938;34:100–108.

[136] Sondheimer EH The mean free path of electrons in metals. Adv Phys. 1952;1(1):1–42.

[137] Brewster MQ Thermal radiative transfer and properties. New York: Wiley; 1992.

[138] Dingle RB The electrical conductivity of thin wires. Proc R Soc Lond Ser A. 1950;201(1067):545–560.

[139] Ziman JM Electrons and phonons: the theory of transport phenomena in solids. New York: Clarendon Press; Oxford University Press; 2001.

[140] Little WA The transport of heat between dissimilar solids at low temperatures. Can J Phys. 1959;37(3):334–349.

[141] Swartz ET, Pohl RO Thermal-resistance at interfaces. Appl Phys Lett. 1987;51(26):2200–2202.

[142] Schelling PK, Phillpot SR, Keblinski P Phonon wave-packet dynamics at semiconductor interfaces by molecular-dynamics simulation. Appl Phys Lett. 2002;80(14):2484–2486.

[143] Zhao H, Freund JB Lattice-dynamical calculation of phonon scattering at ideal Si-Ge interfaces. J Appl Phys. 2005;97(2):024903.

[144] Tian Z, Esfarjani K, Chen G Enhancing phonon transmission across a Si/Ge interface by atomic roughness: first-principles study with the Green’s function method. Phys Rev B. 2012;86(23):235304.

[145] Zhao H, Freund JB Phonon scattering at a rough interface between two fcc lattices. J Appl Phys. 2009;105:1.

[146] Li X, Yang R Effect of lattice mismatch on phonon transmission and interface thermal conductance across dissimilar material interfaces. Phys Rev B. 2012;86(5):054305. 10.1088/0953-8984/24/15/155302 22442141

[147] Kakodkar RR, Feser JP Probing the validity of the diffuse mismatch model for phonons using atomistic simulations. Phys Rev B. 2017;95(12):125434.

[148] Sadasivam S, Waghmare UV, Fisher TS Phonon-eigenspectrum-based formulation of the atomistic Green’s function method. Phys Rev B. 2017;96:17.

[149] Gordiz K, Henry A Phonon transport at interfaces between different phases of silicon and germanium. J Appl Phys. 2017;121:2.

[150] Sakata M, Hori T, Oyake T, et al Tuning thermal conductance across sintered silicon interface by local nanostructures. Nano Energy. 2015;13:601–608.

[151] Wang Z, Alaniz JE, Jang W, et al Thermal conductivity of nanocrystalline silicon: importance of grain size and frequency-dependent mean free paths. Nano Lett. 2011;11(6):2206–2213. 21553856 10.1021/nl1045395

[152] Oyake T, Feng L, Shiga T, et al Ultimate confinement of phonon propagation in silicon nanocrystalline structure. Phys Rev Lett. 2018;120(4):045901. 29437417 10.1103/PhysRevLett.120.045901

[153] Hua C, Chen X, Ravichandran NK, et al Experimental metrology to obtain thermal phonon transmission coefficients at solid interfaces. Phys Rev B. 2017;95(20):205423.

[154] Ju S, Liang X Thermal rectification and phonon scattering in silicon nanofilm with cone cavity. J Appl Phys. 2012;112(5):054312.

[155] Da Cruz CA, Katcho NA, Mingo N, et al Thermal conductivity of nanocrystalline SiGe alloys using molecular dynamics simulations. J Appl Phys. 2013;114:16.

[156] Yang LN, Yang N, Li BW Reduction of thermal conductivity by nanoscale 3D phononic crystal. Sci Rep-Uk. 2013;3. 10.1038/srep01143 PMC3560355 23378898

[157] Ju SH, Liang XG An atomic level investigation of grain-size-dependent thermal conductivity of polycrystalline argon by molecular dynamics. Int J Thermophys. 2014;35(1):32–44.

[158] Zhou YG, Hu M Record low thermal conductivity of polycrystalline si nanowire: breaking the casimir limit by severe suppression of propagons. Nano Lett. 2016;16(10):6178–6187. 27603153 10.1021/acs.nanolett.6b02450

[159] Bird GA Molecular gas dynamics and the direct simulation of gas flows. Oxford New York: Clarendon Press; Oxford University Press; 1994.

[160] Peterson RB Direct simulation of phonon-mediated heat-transfer in a debye Crystal. J Heat Trans T Asme. 1994;116(4):815–822.

[161] Mazumder S, Majumdar A Monte Carlo study of phonon transport in solid thin films including dispersion and polarization. J Heat Transfer. 2001;123(4):749–759.

[162] Lacroix D, Joulain K, Lemonnier D Monte Carlo transient phonon transport in silicon and germanium at nanoscales. Phys Rev B. 2005;72(6):064305.

[163] Jeng M-S, Yang R, Song D, et al Modeling the thermal conductivity and phonon transport in nanoparticle composites using monte carlo simulation. J Heat Transfer. 2008;130(4):042410.

[164] Hao Q, Chen G, Jeng M-S Frequency-dependent Monte Carlo simulations of phonon transport in two-dimensional porous silicon with aligned pores. J Appl Phys. 2009;106(11):114321.

[165] Péraud J-PM, Hadjiconstantinou NG Efficient simulation of multidimensional phonon transport using energy-based variance-reduced Monte Carlo formulations. Phys Rev B. 2011;84(20):205331.

[166] Huang M-J, Kang T-Y A Monte-Carlo study of the phonon transport in nanowire-embedded composites. Int J Ther Sci. 2011;50(7):1156–1163.

[167] Hao Q Influence of structure disorder on the lattice thermal conductivity of polycrystals: A frequency-dependent phonon-transport study. J Appl Phys. 2012;111(1):014309.

[168] Péraud J-PM, Hadjiconstantinou NG An alternative approach to efficient simulation of micro/nanoscale phonon transport. Appl Phys Lett. 2012;101(15):153114.

[169] Jean V, Fumeron S, Termentzidis K, et al Monte Carlo simulations of phonon transport in nanoporous silicon and germanium. J Appl Phys. 2014;115:2.

[170] Péraud J-PM, Landon CD, Hadjiconstantinou NG Monte Carlo methods for solving the Boltzmann transport equation. Annu Rev Heat Transfer. 2014;17:205–265.

[171] Péraud J-PM, Landon CD, Hadjiconstantinou NG Deviational methods for small-scale phonon transport. Mech Eng Rev. 2014;1(2):FE0013–FE0013.

[172] Hua C, Minnich AJ Importance of frequency-dependent grain boundary scattering in nanocrystalline silicon and silicon–germanium thermoelectrics. Semicond Sci Technol. 2014;29(12):124004.

[173] Péraud J-PM, Hadjiconstantinou NG Adjoint-based deviational Monte Carlo methods for phonon transport calculations. Phys Rev B. 2015;91(23):235321.

[174] McGaughey AJH, Jain A Nanostructure thermal conductivity prediction by Monte Carlo sampling of phonon free paths. Appl Phys Lett. 2012;100(6):061911.

[175] Jain A, Yu Y-J, McGaughey AJH Phonon transport in periodic silicon nanoporous films with feature sizes greater than 100 nm. Phys Rev B. 2013;87(19):195301.

[176] Hori T, Shiomi J, Dames C Effective phonon mean free path in polycrystalline nanostructures. Appl Phys Lett. 2015;106(17):171901.

[177] Nomura M, Kage Y, Nakagawa J, et al Impeded thermal transport in Si multiscale hierarchical architectures with phononic crystal nanostructures. Phys Rev B. 2015;91(20):205422.

[178] Parrish KD, Abel JR, Jain A, et al Phonon-boundary scattering in nanoporous silicon films: comparison of Monte Carlo techniques. J Appl Phys. 2017;122:12.

[179] Abbasi MH, Evans JW, Abramson IS Diffusion of gases in porous solids – Monte-Carlo simulations in the knudsen and ordinary diffusion regimes. AlChE J. 1983;29(4):617–624.

[180] Zalc JM, Reyes SC, Iglesia E The effects of diffusion mechanism and void structure on transport rates and tortuosity factors in complex porous structures. Chem Eng Sci. 2004;59(14):2947–2960.

[181] Hori T, Kamino T, Yoshimoto Y, et al Mutual influence of molecular diffusion in gas and surface phases. Phys Rev E. 2018;97(1):013101. 29448343 10.1103/PhysRevE.97.013101

[182] Nakamura Y, Isogawa M, Ueda T, et al Anomalous reduction of thermal conductivity in coherent nanocrystal architecture for silicon thermoelectric material. Nano Energy. 2015;12:845–851.

[183] Cahill DG, Pohl RO Heat-flow and lattice-vibrations in glasses. Solid State Commun. 1989;70(10):927–930.

[184] Zink BL, Pietri R, Hellman F Thermal conductivity and specific heat of thin-film amorphous silicon. Phys Rev Lett. 2006;96(5):055902. 16486955 10.1103/PhysRevLett.96.055902

[185] Cahill DG, Watson SK, Pohl RO Lower limit to the thermal conductivity of disordered crystals. Phys Rev B. 1992;46(10):6131–6140. 10.1103/physrevb.46.6131 10002297

[186] Biswas K, He J, Wang G, et al High thermoelectric figure of merit in nanostructured p-type PbTe–MTe (M = Ca, Ba). Energy Environ Sci. 2011;4(11):4675.

[187] Kim W, Majumdar A Phonon scattering cross section of polydispersed spherical nanoparticles. J Appl Phys. 2006;99(8):084306.

[188] Mingo N, Hauser D, Kobayashi NP, et al “Nanoparticle-in-Alloy” approach to efficient thermoelectrics: silicides in SiGe. Nano Lett. 2009;9(2):711–715. 19128146 10.1021/nl8031982

[189] Zhang H, Minnich AJ The best nanoparticle size distribution for minimum thermal conductivity. Sci Rep-Uk 2015 p. 5. 10.1038/srep08995 PMC4355732 25757414

[190] Zuckerman N, Lukes JR Acoustic phonon scattering from particles embedded in an anisotropic medium: A molecular dynamics study. Phys Rev B. 2008;77(9):094302.

[191] Ju SH, Liang XG Detecting the phonon interference effect in Si/Ge nanocomposite by wave packets. Appl Phys Lett. 2015;106(20).

[192] Feng L, Shiga T, Han H, et al Phonon-interference resonance effects by nanoparticles embedded in a matrix. Phys Rev B. 2017;96(22):220301.

[193] Davis BL, Hussein MI Nanophononic metamaterial: thermal conductivity reduction by local resonance. Phys Rev Lett. 2014;112(5):055505. 24580612 10.1103/PhysRevLett.112.055505

[194] Honarvar H, Yang L, Hussein MI Thermal transport size effects in silicon membranes featuring nanopillars as local resonators. Appl Phys Lett. 2016;108:26.

[195] Iskandar A, Gwiazda A, Huang Y, et al Modification of the phonon spectrum of bulk Si through surface nanostructuring. J Appl Phys. 2016;120:9.

[196] Ma D, Ding H, Meng H, et al Nano-cross-junction effect on phonon transport in silicon nanowire cages. Phys Rev B. 2016;94(16):165434.

[197] Xiong S, Sääskilahti K, Kosevich YA, et al Blocking phonon transport by structural resonances in alloy-based nanophononic metamaterials leads to ultralow thermal conductivity. Phys Rev Lett. 2016;117(2):025503. 27447516 10.1103/PhysRevLett.117.025503

[198] Honarvar H, Hussein MI Two orders of magnitude reduction in silicon membrane thermal conductivity by resonance hybridizations. Phys Rev B. 2018;97(19):195413.

[199] Han H, Potyomina LG, Darinskii AA, et al Phonon interference and thermal conductance reduction in atomic-scale metamaterials. Phys Rev B. 2014;89(18):180301.

[200] Han HX, Feng L, Xiong SY, et al Effects of phonon interference through long range interatomic bonds on thermal interface conductance. Low Temp Phys. 2016;42(8):711–716.

[201] Han H, Feng L, Xiong S, et al Long-range interatomic forces can minimize heat transfer: from slowdown of longitudinal optical phonons to thermal conductivity minimum. Phys Rev B. 2016;94(5):054306.

[202] Kosevich YA Multichannel propagation and scattering of phonons and photons in low-dimension nanostructures. Phys Usp. 2008;51(8):848–859.

[203] Dieb TM, Ju S, Yoshizoe K, et al MDTS: automatic complexmaterials design using Monte Carlo tree search. Sci Technol Adv Mater. 2017;18(1):498–503. 28804525 10.1080/14686996.2017.1344083 PMC5532970

[204] Ju S, Shiga T, Feng L, et al Designing nanostructures for phonon transport via bayesian optimization. Phys Rev X. 2017;7(2):021024.

[205] Yamawaki M, Ohnishi M, Ju S, et al Multifunctional structural design of graphene thermoelectrics by Bayesian optimization. Sci Adv. 2018;4:6. 10.1126/sciadv.aar4192 PMC6003749 29922713

